# Analytical Validation of MINI-PET as Point-of-Care for Erythrocyte Sedimentation Rate Measure in Horses

**DOI:** 10.1155/2023/9965095

**Published:** 2023-11-15

**Authors:** Carolina Pieroni, Andrea Grassi, Marianna Pantoli, Mirko Berretti, Stefano Messina, Chiara Giovannini, George Lubas, Daniela Diamanti

**Affiliations:** ^1^DIESSE- Diagnostica Senese S.p.A. Società Benefit, Strada dei Laghi, 35-39, Monteriggioni, Siena, Italy; ^2^I-Vet Diagnostica Veterinaria, Via Ettore Majorana, 10, Flero, Brescia, Italy; ^3^Istituto Zooprofilattico Sperimentale della Lombardia e dell'Emilia Romagna, Sede Territoriale di Pavia, Str. Privata Campeggi, 59, Pavia, Italy; ^4^Azienda Ospedaliera Universitaria Senese, V.le Mario Bracci, 11, Siena, Italy; ^5^Il Ceppo Equine Hospital, Strada Monteresi 3, Monteriggioni, Siena, Italy; ^6^Clinica Veterinaria Colombo, VetPartners Italia, V.le Colombo 153, Lido di Camaiore, Lucca, Italy

## Abstract

The erythrocyte sedimentation rate (ESR) is a widely used diagnostic assay in human medicine but nowadays poorly applied in veterinary medicine. This test measures the speed (millimeters per hour) at which red blood cells settle in a whole anticoagulated blood tube. In human medicine, high ESR values are associated with various disorders, including infections, rheumatoid arthritis, oncologic diseases, and other inflammatory conditions. The ESR can also be influenced by some factors such as age and gender. In veterinary medicine, the ESR with the Westergren manual method was almost forgotten over the years due to blood consumption and long turn-around time. The instrument MINI-PET, using a modified Westergren method, does not require blood consumption or release waste product and recently has been applied in canine medicine. The aims of the study in the horse were as follows: to establish the appropriate time to read the ESR with the Westergren reference method; to compare the MINI-PET ESR results with the reference technique; to assess the ESR reference intervals with MINI-PET; and to establish the ESR stability from collection at different time points by MINI-PET. Using 150 horses, we established 60 minutes as the appropriate time for ESR reading with the Westergren method. Moreover, ESR results obtained in 8 minutes with MINI-PET showed a good correlation with the Westergren ESR. Reference intervals (RIs) with MINI-PET were established in mm/h for the healthy horses (geldings 18.6–100.1; stallions, 13.8–55.7; and mares 1–73.7) according to the American Society of Veterinary Clinical Pathology guidelines. In addition, the ESR stability from the blood collection time was evaluated in the MINI-PET on 15 horses: at room temperature, ESR is stable up to 8 hours and at 4°C up to 24 hours. In conclusion, MINI-PET represents a rapid and reliable tool for measuring ESR in horses, offering a valid option to replace the traditional manual technique.

## 1. Introduction

The erythrocyte sedimentation rate (ESR) is a widely used diagnostic assay in human medicine, especially in inflammatory conditions [1–3]. This test expresses the speed (mm/h) at which red blood cells settle in a whole anticoagulated blood tube [4], measuring the resulting plasm column.

During an inflammatory process, the plasma levels of several acute-phase proteins, such as fibrinogen and immunoglobulins, increase. These proteins have a positive charge, and their elevated concentration in the plasma is able to neutralize the negative charge on the surface of red blood cells. This leads to an increase in blood viscosity and facilitates the erythrocytes' aggregation [2].

In human patients, high ESR values can be associated with various disorders, including infections, rheumatoid arthritis, oncologic diseases, anemia, obesity, renal disorders, collagen vascular diseases, red blood cell abnormalities, and other inflammatory conditions [1, 5, 6]. Moreover, ESR values can be influenced by numerous paraphysiological factors. Higher ESR levels are observed in female patients compared to males, and they are also associated with increasing age, smoking, and pregnancy. On the other hand, factors such as altitude, physical activity, and low alcohol consumption are linked to a decrease in ESR levels [7].

The ESR measurement can also serve as a valuable diagnostic tool in veterinary medicine. In the past, manual methods such as Westergren or Wintrobe have been utilized to investigate ESR in horses, cats, dogs, and sheep [8–10].

However, there has been a declining trend in the ESR evaluation over the years, likely due to the significant time required for the manual test performance and the additional blood consumption due to the use of sodium citrate as blood anticoagulant.

Similar patterns can be observed in scientific literature reporting ESR. Studies on ESR on horses started in the 1930s and extended mainly until the 1990s [11]. The limited available literature indicates that ESR values in horses are higher compared to those in humans [12] and the equine ESR can be influenced by some conditions such as exercise or breed as well as in humans [7, 12–15].

Moreover, the handling of the Westergren reference method for ESR determination in horses remains controversial, considering that different studies have collected ESR values at varying time points ranging from 10 minutes [16] to 60 minutes [12, 14, 17], without clear evidence supporting a definitive choice to set up the time point.

MINI-PET (DIESSE Diagnostica Senese, S.p.A., Società Benefit) is an automatic instrument for the determination of the ESR specifically in veterinary medicine. The employment of MINI-PET has been already assessed in dogs, where ESR reference intervals have been defined in nonanemic animals [10, 18].

The aims of this study arranged in the horse were as follows: (1) to establish the appropriate time to read the ESR with the Westergren reference method; (2) to compare the ESR results obtained with both methods; (3) to assess the ESR reference intervals with MINI-PET; and (4) to investigate the ESR stability in EDTA tubes at different time postcollection using MINI-PET.

## 2. Materials and Methods

### 2.1. Horse Blood Samples' Collection and Grouping

A population of 150 horses from various regions in Northern and Central Italy, consisting of both healthy and diseased animals, was included in ESR surveys. The study was performed on left-over samples for routine diagnostical purposes, and for this reason, a formal ethical approval was not necessary. One hundred and ten horses were identified as clinically healthy based on their medical history, physical examination, and complete blood count. Animals in apparently clinically normal status showing hematocrit and hemoglobin values outside the reference interval (*n* = 16) and pregnant females (*n* = 6) were excluded from the healthy group, as these conditions can affect normal ESR levels [19]. Ten (*n* = 10) horses were affected by various diseases, including colic (*n* = 2), pulmonary hemorrhage (*n* = 1), septic arthritis (*n* = 1), foot infection (*n* = 1), cough (*n* = 2), pneumonia (*n* = 2), and equine motor neuron disease (*n* = 1). Eight (*n* = 8) horses were not included in the healthy group as the veterinarian in charge reported that four had a recent castration, one was anorectic, and three were unhealthy due to unspecified health issues at the time of investigation (see Table 1).

Blood samples were taken from the jugular vein and collected in 3.0 ml K3-EDTA tubes (Vacutest KIMA, Arzergrande, Padua, Italy) in the early morning hours, with the animals at rest, following the protocol outlined by Dalton [14]. Timing from the blood collection to the ESR determination varied according to the different type of arranged study.

To validate the choice of 60 minutes as the time for ESR determination by the Westergren reference method and for the comparison between MINI-PET and Westergren, the same heterogeneous group of 150 horses was considered. This population included colts, mares, stallions, and geldings belonging to different breeds, both sick and healthy, ranging in age from 1 to 27 years. Blood samples were analyzed within two hours of blood collection.

To establish the ESR reference intervals, only healthy horses (*n* = 110) were included, subtyped the population into three groups: mares (*n* = 38), stallions (*n* = 20), and geldings (*n* = 52). These horses were defined healthy based on physical examination and hematological profile. The ESR measurement was performed within two hours of blood sampling. Colts were not included due to their limited number (only four).

The sample stability for the ESR test was assessed on a different group of horses (*n* = 15), from which two blood samples were drawn and analyzed within one hour of collection, the minimum time necessary from sampling to the analysis in the laboratory.

### 2.2. Complete Blood Count

The same blood tube, used for measurement of ESR with MINI-PET, was processed to carry out the complete blood count (CBC) using Siemens ADVIA 2120 instrument and the preparation of a blood smear, which was automatically stained (Siemens Hematek 3000). The blood smears were examined microscopically to evaluate erythrocytes, leucocytes, and platelets and the possible presence of haemoparasites.

### 2.3. Description of the Westergren Reference Test

The reference test selected to measure the ESR was the manual Westergren method [4, 20]. Samples of 150 horses were analysed within two hours from blood collection. In 0.25 ml of sodium citrate, 1.0 ml of blood collected on a K3-EDTA tube was added to set a ratio of 1 : 4. The tubes were mixed by inversion and kept under continuous agitation for few minutes. The Westergren method was then performed within 2 hours from the sampling. The ESR determination was carried out using glass pipettes with an internal diameter of 2.5 mm “takives” (FL Medical s.r.l, Torreglia, Padua, Italy), following the guidelines set by the International Council for Standardization in Haematology (ICSH) 1993 [4]. The ESR measure was recorded every five minutes, and the result was collected at 5, 10, 15, 20, 25, 30, 35, 40, 45, 50, 55, and 60 minutes.

### 2.4. ESR Comparison between Westergren and MINI-PET Methods

Blood samples were analysed within two hours from collection, stored at room temperature, away from light and heat sources. Before performing the Westergren manual method, the ESR was assessed initially using the MINI-PET device. This choice was made because the MINI-PET does not consume blood and does not require any particular handling of the sample. The device is capable of continuously loading, and it can analyse up to four EDTA blood samples simultaneously, utilizing an optical system [10]. The samples were manually and gently resuspended by mixing samples for 10 times and then introduced into the MINI-PET for automatic ESR determination based on the modified Westergren method [21]. The results were obtained in 8 minutes without any sample manipulation, consumption, or waste production. The 8 minutes reading time was assessed during the analytical validation setting in DIESSE. Other time points have been investigated, but they did not meet the statistical criteria of validity (data were not shown).

### 2.5. Interassay Precision

The MINI-PET precision has been determined with “Sedicheck” (REF:10441/PET, DIESSE Diagnostica Senese S.p.A.). It is a ready-to-use quality control (QC) material composed by stabilized suspension of photo-absorbing particles in a specific preservative material. There are two levels (level 1 and level 2) of control material that represents the ESR values that can be obtained: level 1 is a low value, while level 2 corresponded to a higher value than previous. The QC was used in 5 replicates both in level 1 and level 2. All QC samples were analyzed 2 times a day for 5 days, by one operator using one instrument, obtaining 50 determinations for each level.

### 2.6. Reference Interval

Reference intervals (RIs) were evaluated following the American Society for Veterinary Clinical Pathology (ASVCP) guidelines, in the 110 healthy horses. The horses were divided into three groups: A (geldins *n* = 52), B (stallions *n* = 20), and C (mares *n* = 38). For each of the three groups, the RI has been calculated with the results obtained at 8 minutes from the MINI-PET analysis.

### 2.7. Equine Blood Sample Stability

To assess the stability, the ESR was measured with MINI-PET immediately within one hour from the sample collection (T0). One duplicate sample tube was employed for ESR determination at 2, 4, 6, 8, and 24 hours from T0, storing it at room temperature (20–25°C). The other duplicate sample was refrigerated at 4°C until the following day (24 h); in this case, before measuring the ESR with the MINI-PET, the sample was placed at room temperature for 30 minutes.

### 2.8. Statistical Analysis

The appropriate time to read the ESR Westergren reference technique was established by Passing and Bablok regression analysis [22], considering different time points at 5, 10, 15, 20, 25, 30, 35, 40, 45, 50, 55, and 60 minutes.

This test combined with Bland–Altman analysis [23] was applied to compare ESR results from the Westergren reference technique acquired at 60 minutes and MINI-PET. Nonparametric test of Spearman and the rank coefficient correlation (*ρ*) were reported to assess the strength of the association between the two methods.

For interassay precision, for each level, the mean and standard deviation (SD) from all the results obtained over the 5 days of experiment were computed and interassay coefficient of variation (CV%) was calculated.

Following the ASVCP reference interval guidelines [24], data of distributions were reported (Gaussian). A parametric method based on normal distribution (Kolmogorov–Smirnov test) with 90% of confidence interval of the reference limits calculated lower and upper reference limits for geldings and stallions (using mean and standard deviation). On the contrary, a nonparametric method (percentile) was applied for mares to calculate lower and upper reference limits (median, lower, and higher limit).

For stability study, multiple variable graphs, including Box-and-Whisker plots, and Friedman test were used to compare ESR at T0 with measures at 2, 4, 6, 8, and 24 hours at room temperature and after 24 hours stored at 4°C.

Statistical elaborations were performed using MedCalc software, version 20.113-64-bit (Mariekerke, Ostend, Belgium). Qualitative plots are also obtained in Python with matplotlib 3.5.1.

## 3. Results

### 3.1. Validation of ESR with Westergren Reference Method at 60 Minutes

**Figure 1:**
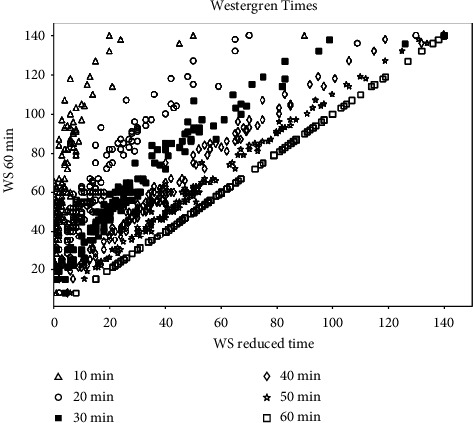
Sedimentation level obtained at standard sedimentation time (60 min) vs. the sedimentation level obtained at 10 min, 20 min, 30 min, 40 min, and 50 min.

To investigate time points shorter than 60 minutes for method comparison with the Westergren reference method, ESR values were recorded every five minutes at 5, 10, 15, 20, 25, 30, 35, 40, 45, 50, 55, and 60 minutes.  Figure 1 shows the relation between the sedimentation level obtained at standard reference time (60 minutes) vs. the sedimentation level obtained at shorter times (only 10, 20, 30, 40, 50, and 60 minutes reading times were plotted).

Passing and Bablok regression analysis was applied to evaluate the linear relationship between ESR at 60 minutes and ESR at each time point as reported in Table 2. Starting from 40 minutes, results were close to proportionality, increasing the slope *b* as showed by 95% CI of *b* (*y* *=* *a* *+* *bx*); however, only outcomes at 55 minutes were perfectly overlapping with those at 60 minutes, although also lower time points produced a very high correlation. Negative values for the intercept *a* highlighted a negative constant between the two time points.

### 3.2. Method Comparison for ESR between Westergren Reference and MINI-PET Methods

**Figure 2:**
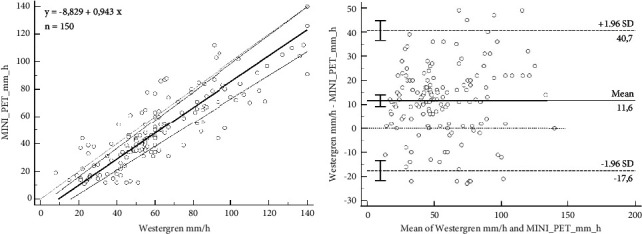
(a) Passing and Bablok regression analysis between the measurement of ESR with the Westergren reference method and MINI-PET. (b) Bland–Altman analysis between the measurement of ESR with the Westergren reference method and MINI-PET.

For the comparison between the reference and MINI-PET methods, 150 samples from healthy and sick horses were enrolled. In the Passing and Bablok analysis of regression, the intercept of systematic differences was −8.83 (95% CI: −15.00 to −4.11) and the slope proportional differences was 0.94 (95% CI: 0.86 to 1.10), confirming the two methods were proportional as reported in Figure 2(a).

The Bland–Altman analysis showed a positive bias of 11.56 between the two tests (95% CI: 9.16 to 14.00) with upper limit of 40.67 (95% CI: 36.57 to 44.78) and lower limit of −17.55 (95% C: −21.66 to −13.45), as shown in Figure 2(b).

The ESR measurement between MINI-PET and Westergren reference method showed a good correlation coefficient with the Spearman test (*ρ* = 0.86; 95% CI: 0.82 to 0.90; *p*  <  0.0001).

### 3.3. Evaluation of Interassay Precision

The precision tests were performed on the MINI-PET using the ESR quality control Sedicheck, for 5 consecutive days, calculating for each level the mean, SD, and CV%. The mean obtained for level 1 was 11 mm/h (min value: 8 mm/h; max value: 12 mm/h), and the CV% was 9.8% (SD: 1.08). For level 2, the mean was 62 mm/h (min value: 58 mm/h, max value: 65 mm/h), and the CV% was 2.7% (SD: 1.67).

### 3.4. Proposed Reference Interval (RI) for Equine ESR Using MINI-PET

**Figure 3:**
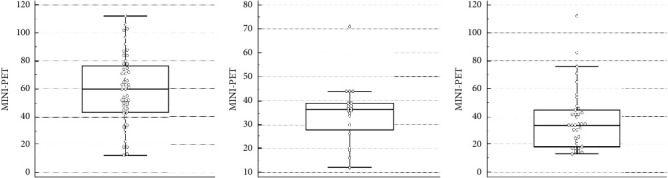
(a) Box and Whisker graph for ESR RI in geldings, arithmetic mean value = 59.3 mm/h, and standard deviation = 24.8 mm/h; (b) Box and Whisker graph for ESR RI in stallions, arithmetic mean value = 34.8 mm/h, and standard deviation = 12.7 mm/h; (c) Box and Whisker graph for ESR RI in mares, median value = 34.0 mm/h, lower limit 13.9 mm/h, and upper limit 87.3 mm/h.

**Figure 4:**
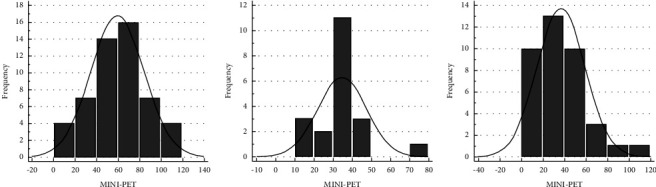
(a) Gaussian distribution of ESR RI in geldings; (b) Gaussian distribution of ESR RI in stallions; and (c) nonparametric distribution of ESR in mares.

In group A, including 52 geldings, the ESR RI ranged between 18.6 mm/h (90% CI: 8.7 to 28.4) and 100.1 mm/h (90% CI: 90.3 to 110.0), with a mean of 59.3 mm/h (SD: 24.8). Histograms and graphs of the RI were reported in Figures 3(a) and 4(a).

Group B composed by 20 stallions generated a RI ranged between 13.8 mm/h (90% CI: 5.6 to 22.1) and 55.7 mm/h (90% CI: 47.4 to 63.9), with a mean of 34.8 mm/h (SD: 12.7), as shown in Figures 3(b) and 4(b)).

The RI for group C consisted of 38 mares ranged between 13.9 mm/h and 87.3 mm/h (median: 34.0 mm/h; lowest value 13.0 mm/h and highest value 112.0 mm/h) as shown in Figures 3(c) and 4(c).

### 3.5. Sample Stability for Equine ESR with MINI-PET

**Figure 5:**
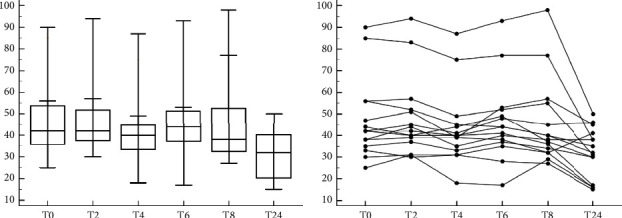
(a): Evaluation of stability for ESR test in blood samples stored at room temperature and at different times. In the Box and Whisker graph in the *x* axis is reported the time in hour and in the *y* axis is reported the ESR (mm/h). (b): Evaluation of stability for ESR test in blood samples stored at room temperature and at different times. In the Friedman test in the *x* axis is reported the time in hour, in the *y* axis is reported the ESR (mm/h).

The first set of samples was kept at room temperature and was analysed within an hour of collection and then at 2, 4, 6, 8, and 24 hours. It was observed that horses' ESR remained stable for up to 8 hours and decreased significantly at 24 hours as shown in Figures 5(a) and 5(b).

**Figure 6:**
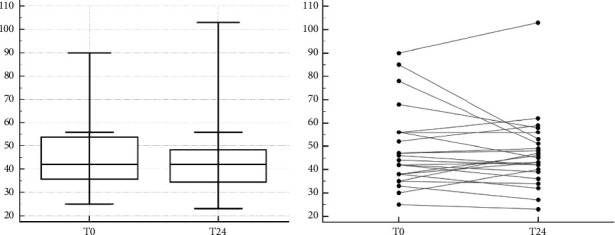
(a): Evaluation of stability for the ESR test in blood samples stored at 4°C for 24 hours. In the Box and Whisker graph in the *x* axis is reported the time in hour and in the *y* axis is reported the ESR (mm/h). (b): Evaluation of stability for the ESR test in blood samples stored at 4°C for 24 hours.

The set of refrigerated samples did not show significant difference between T0 and T24 ESR values (*P* = 0.402) as shown in Figures 6(a) and 6(b).

## 4. Discussion

The erythrocyte sedimentation rate is a commonly used laboratory test in humans to assess the presence of many pathological conditions such as inflammation [1, 25] though it is affected by various factors [7, 12–15]. In horses, ESR was widely engaged to evaluate the presence of infections or inflammatory disorders [26] and to monitor the physiological status before and after a race or a training as well as the time used to return to normal ESR values. These fluctuations have been explained by some investigations showing how the exercise in horse can induce changes in both hematocrit and ESR levels, specifically an increase during physical activity and a decrease during rest [12, 14]. Thereby, based on literature evidences, all the blood equine samples in this study were collected in the morning to minimize the impact of physical activity on ESR results.

Nowadays, the Westergren method has been still considered the gold standard for the evaluation of ESR humans, according to the ICSH, measuring ESR at 60 minutes. This manual method applied to horses has a long turn-around time (TAT) and is influenced by many variables, so the ESR test is not currently used. The implementation of an automatic device as MINI-PET based on the modified Westergren method could represent a faster and less laborious option than the manual method. MINI-PET measures the ESR in 8 minutes in the same EDTA blood tubes intended for the blood cell count and allows the simultaneous analysis of 4 samples without blood consumption, waste production, and without an additional withdrawal for the ESR test.

A review of the literature revealed a discrepancy in the timing of the acquisition of Westergren measure among authors [12, 14, 16, 17]. To solve this issue, in this work, the ESR was recorded at 5-minute intervals up to 60 minutes during the Westergren method running. The Passing and Bablok test was employed to assess the appropriate time point for ESR result acquisition of the manual method. The analysis identified that only values at 55 minutes respected all statistical criteria and there was a poor correlation with lower time points, even used in previous papers [12, 14, 16, 17]. As reported in Table 2, especially at 20 and 30 minutes, the obtained data failed the Passing and Bablok regression equation even if the correlation coefficient showed that these time points could be acceptable. Starting from 40 minutes, results were close to proportionality, increasing the slope *b*, but only outcomes at 55 minutes were perfectly overlapping with those at 60 minutes. The data in the Table 2 showed that it was not statistically justifiable to use Westergren values less than 55–60 minutes. To our knowledge, for the first time, this investigation validated Westergren data at 60 minutes in horses as well as already adopted in humans.

The analytical performance of MINI-PET, compared to the standard Westergren technique, was also evaluated. The good Spearman's correlation (*ρ* = 0.86) validated MINI-PET in equine medicine in replacement of the manual method, confirmed by slope 95% CI that included value one, ensuring proportionality between the two methods.

The interassay precision of MINI-PET was evaluated using Sedicheck both in level 1 and level 2 over 5 days and twice a day. The obtained imprecision was <10% for both levels (CV% level 1: 9.8%, CV% level 2: 2.7%), highlighting excellent result reproducibility.

The stability of equine blood samples in EDTA tubes for ESR assay by MINI-PET was investigated. To evaluate the ESR trend at room temperature, the test was performed every two hours from the sampling. The value remained constant up to 8 hours, instead at 24 hours started to decrease significantly. Samples analyzed the following day after storage for 24 hours at 4°C showed no significant difference in T0-T24 ESR values. This investigation proved that if it is not possible to perform the ESR test within 8 hours from the collection, the sample must be stored at 4°C up to 24 hours, allowing the ESR measure by MINI-PET in a different moment from the withdrawn. This evaluation has been already conducted by Gori in dogs and cats [18].

Finally, using MINI-PET, we proposed ESR reference intervals for healthy horses considering separately stallions (mean 34.8 ± SD 12.7), mares (median 34.0, range 13.9–87.3), and geldings (mean 59.3 ± SD 24.8). It should be noted that RIs for stallions and mares were almost overlapping each other and in mares were little wider. Investigations of the ESR normal level in different horse populations have been already performed. Ju [15] and Lumsden [19] both used the Wintrobe method to set-up ESR reference ranges in stallions (mean 17.4 ± SD 6.4), geldings (mean 43.2 ± SD 3.0), mares (mean 45.0 ± SD 2.9), and thoroughbred mares (mean 49.3 ± SD 6.4) and ponies (mean 54 ± SD 4.2). Wood [12] used the Westergren method and studied the ESR in healthy horses before and after race, suggesting potential RI for geldings (prerace: mean 50.0 ± SD 15.2; postrace: mean 15.7 ± SD 15.4) and mares (prerace: mean 52.4 ± SD 12.3; postrace: mean 11.6 ± SD 8.1). The MINI-PET values are mismatched from those proposed by Ju [15] or by Lumsden [19] probably for the different tools (Wintrobe vs. Westergren original or modified, as MINI-PET), and from those from Wood [12] perhaps for the reduced enrollment number of animals. Even if this work suggested possible RI for some horse populations, additional investigations should be conducted to define more specific and appropriate intervals, by creating larger and homogeneous groups including ponies.

Moreover, this research confirmed that healthy horses have higher ESR than humans, as already showed in the literature, indeed in the horses ESR (ca. 50 mm/h) was much higher than in humans (ca 10 mm/h) [12, 14, 27]. Although the smaller RBCs and the lower concentration of fibrinogen [12] are factors linked to lower human ESR [1, 28], in the horses, these conditions are not enough to decrease the ESR, probably for the presence of some unknown plasma factors that makes erythrocytes more flexible and thus facilitates the aggregation [29].

## 5. Conclusion

Despite its low sensitivity and low specificity to discover specific diseases, the friendly and easy accomplishment and low cost execution can make the ESR an attractive new test in equine clinical practice. The good correlation between MINI-PET results and the gold standard Westergren method supported the effectiveness of MINI-PET as a rapid and reliable tool in equine veterinary medicine. Moreover, the evaluation of equine blood samples stability for ESR test allows the test to be run away from the farm.

## Figures and Tables

**Table 1:** Horses' population investigated grouped as breed, healthy or sick, mares, stallions, or geldings.

Horse breeds	Sick mares (*N*. 19)	Healthy mares (*N*. 38)	Sick stallions (*N*. 11)	Healthy stallions (*N*. 20)	Sick geldings (*N*. 10)	Healthy geldings (*N*. 52)
Percheron						3
Italian Trotter		1	1			
Belgian Workhorse			1		1	14
TPR						8
Anglo-Arabian		1		2		4
Argentine	1					2
Frisian		1	1		1	
KWPN	2	1		1		
Paint	1					
Thoroughbred	6	5		2		2
Quarter Horse		2		1		2
Dutch Workhorse				1		
Belgian saddle			1			
Italian saddle	4	2	1		3	8
Pony of Esperia	2	2	1	1		1
Quarab				1		
French saddle					1	1
Pony Exmoor		1				
Andaluso/PRE			3	4		1
Unknown	1			1	1	1
German saddle	2	2	1	4	3	5
Poland saddle				1		
French Trotter		20				
Cruzado			1	1		

**Table 2:** Passing and Bablok regression equation and relative confidence interval (“*a*” means 95% CI for intercept *a*; “*b*” means 95% CI for slope *b*).

Timing comparison	Regression equation	Correlation coefficient (*ρ*)	95% CI (*ρ*)
60 min vs 5 min	—	0.38	0.23 to 0.51
60 min vs 10 min	*y* = −3.08 + 0.11*x* (*a*: −3.92 to −2.27; *b*: 0.09 to 0.12)	0.82	0.76 to 0.87
60 min vs 15 min	*y* = −7.88 + 0.28*x* (*a*: −9.46 to −6.25; b: 0.25 to 0.31)	0.92	0.89 to 0.94
60 min vs 20 min	*y* = −11.44 + 0.44*x* (*a*: −13.72 to −9.00; *b*: 0.40 to 0.48)	0.96	0.94 to 0.97
60 min vs 25 min	*y* = −13.63 + 0.58*x* (*a*: −16.05 to −11.19; *b*: 0.54 to 0.62)	0.97	0.96 to 0.98
60 min vs 30 min	*y* = −14.20 + 0.70*x* (*a*: −16.74 to −11.92; *b*: 0.66 to 0.74)	0.98	0.97 to 0.99
60 min vs 35 min	*y* = −12.75 + 0.78*x* (*a*: −14.96 to −10.63; *b*: 0.75 to 0.82)	0.99	0.99 to 0.99
60 min vs 40 min	*y* = −11.58 + 0.86*x* (*a*: −13.15 to −9.71; *b*: 0.83 to 0.90)	0.99	0.98 to 0.99
60 min vs 45 min	*y* = −9.46 + 0.92*x* (*a*: −10.76 to −7.95; *b*: 0.89 to 0.94)	0.99	0.99 to 1.00
60 min vs 50 min	*y* = −6.68 + 0.96*x* (*a*: −7.93 to −5.65; *b*: 0.94 to 0.98)	0.99	0.99 to 1.00
60 min vs 55 min	*y* = −4.00 + 1.00*x* (*a*: −4.00 to −2.87; *b*: 0.98 to 1.00)	1.00	1.00 to 1.00

Spearman correlation coefficient calculated between 60 min respect to each time point. For time at 5 minutes, the Passing and Bablok regression procedure was not reported as the linear model validity was not established by the software.

## Data Availability

The datasets used during this study are available from the corresponding authors upon reasonable request.
